# Scalp trichilemmal cyst: A case report

**DOI:** 10.1097/MD.0000000000034213

**Published:** 2023-07-14

**Authors:** Zipeng Zhu, Xiaoqian Yang, Jinyue Fu, Shubao Zhang, Zonglei Chong

**Affiliations:** a Department of Neurosurgery, Liaocheng People’s Hospital, Liaocheng, Shandong, China; b Department of Neurology, Liaocheng People’s Hospital, Liaocheng, Shandong, China; c Department of Neurosurgery, Liaocheng Dongchangfu People’s Hospital, Liaocheng, Shandong, China.

**Keywords:** clinical characteristics, diagnosis and treatment

## Abstract

Trichilemmal cyst (TC), also known as trichodermal cyst, trichodermal isthmus-degenerative cyst. It is a benign skin lesion originating from the outer hair root sheath, with low incidence and few reports.

**Patient concerns:**

A 41-year-old patient had found a scalp lump for more than 10 years. A 2.0 cm × 1.0 cm × 1.0 cm lump on the right occipital region was touched more than 10 years ago without special treatment. In the past 2 years, the lump has gradually increased. Physical examination: 4 protruding lumps can be reached in the scalp. One lump in the right occipital region is about 3.0 cm × 2.0 cm × 2.0 cm, with 1 lump immediately below and 2 lumps in the left temporal region. All lumps can be pushed.

**Diagnoses:**

The lesion is located in dermis, The lesion is solid, and the contents of the cyst were cheese-like white material, and the inner and outer walls of the cyst were smooth and shiny. Pathological results showed that the lesion was TC. The cyst wall is epidermal tissue, the spinous layer and basal layer are intact, there is no granular layer, and the protein in the cyst is dense.

**Interventions:**

All lumps were completely surgically removed.

**Outcomes:**

The wound healed well after TC resection. There was no recurrence of TC after 1 year follow-up.

**Lessons:**

The clinical manifestations of scalp TC are not specific, and the diagnosis needs pathological examination, and the prognosis of total excision is good.

## 1. Introduction

Trichilemmal cyst (TC), also known as trichodermal cyst, trichodermal isthmus-degenerative cyst. It is a benign skin lesion originating from the outer hair root sheath, with low incidence and few reports.^[1,2]^ A case of TC was diagnosed and treated in our department, and the report is as follows.

## 2. Case presentation

**Figure 1.**
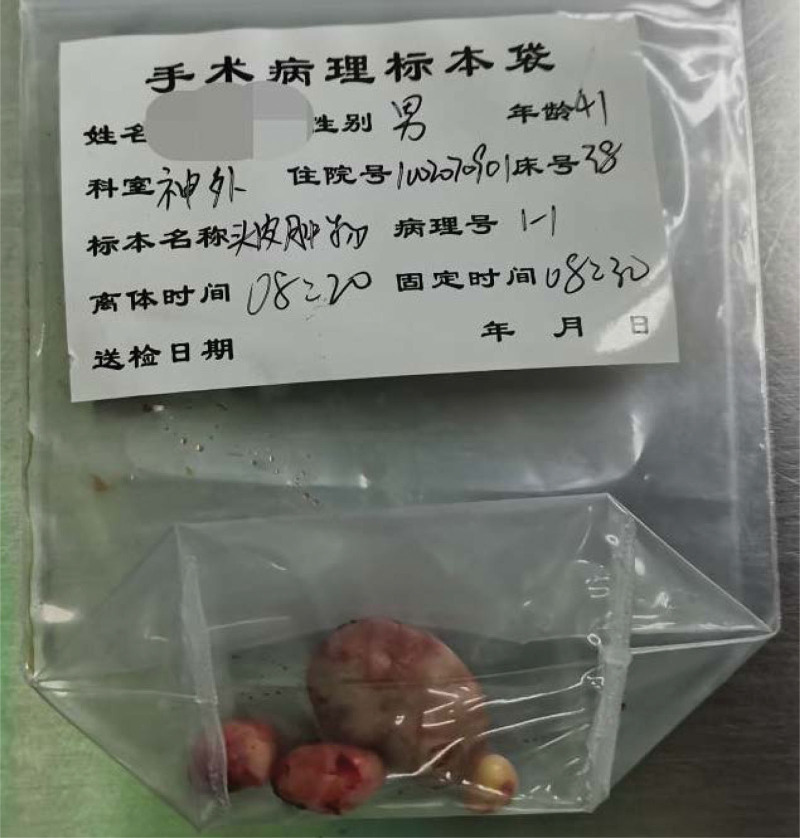
There are 4 pathological specimens with smooth and complete cyst.

**Figure 2.**
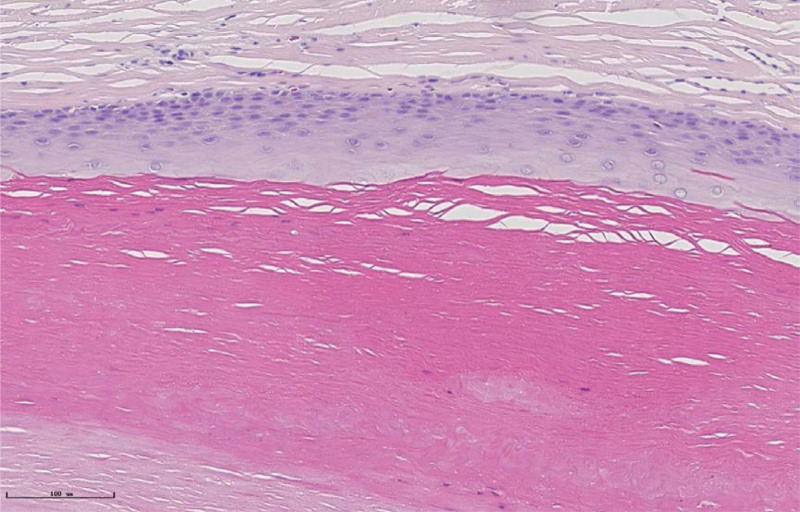
The pathological section was stained by routine method. The wall of the cyst was epidermal tissue without granular layer, and the contents of the cyst were dense keratin.

**Figure 3.**
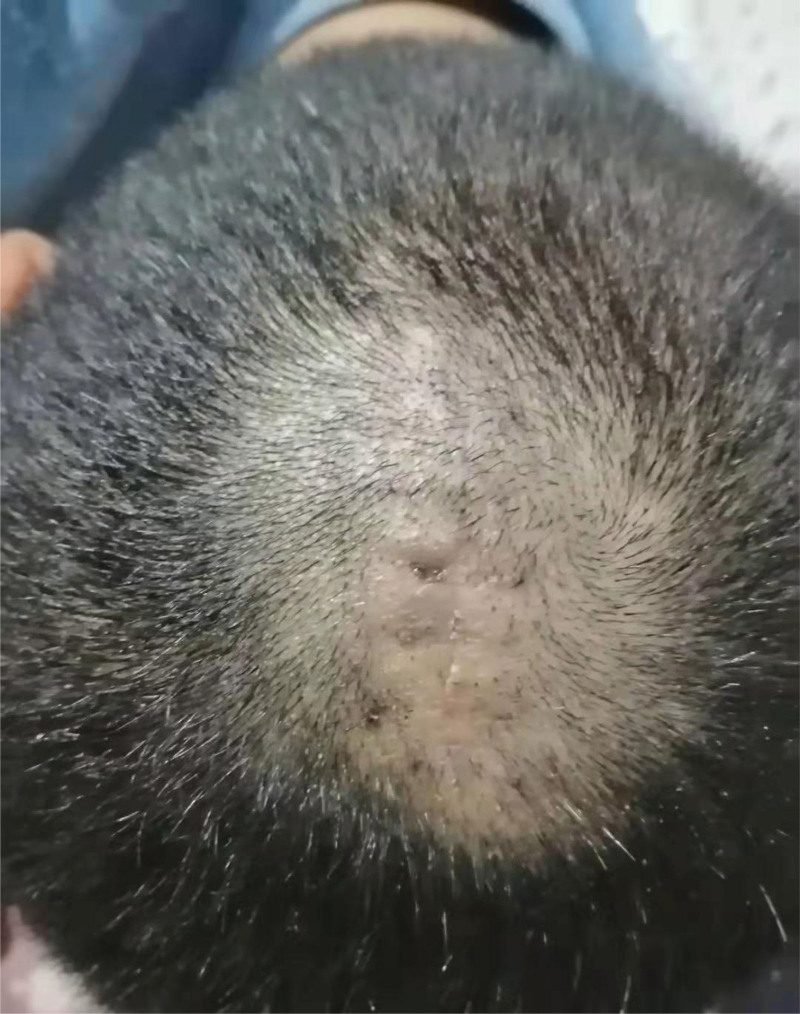
Postoperative follow-up showed that the incision healed well and there was no recurrence.

A 41-year-old patient was admitted to our hospital because he had found a scalp lump for more than 10 years. A 2.0 cm × 1.0 cm × 1.0 cm lump on the right occipital region was touched more than 10 years ago without special treatment. In the past 2 years, the lump has gradually increased. Physical examination: 4 protruding lumps can be reached in the scalp. One lump in the right occipital region is about 3.0 cm × 2.0 cm × 2.0 cm, with 1 lump immediately below and 2 lumps in the left temporal region. All lumps can be pushed, with no tenderness, no wave motion, normal surface hair, no redness and ulceration. Local anesthesia with lidocaine was given during the operation; The lesion is located in dermis, and the boundary with epidermis is clear; The lesion is solid, the cyst wall is smooth and complete, there is no obvious blood supply vessel, and the boundary with surrounding tissues is clear. All lumps were completely surgically removed, and the contents of the cyst were cheese-like white material, and the inner and outer walls of the cyst were smooth and shiny (Fig. 1). Pathological results showed that the lesion was TC. The cyst wall is epidermal tissue, the spinous layer and basal layer are intact, there is no granular layer, and the protein in the cyst is dense (Fig. 2). The wound healed well after TC resection (Fig. 3). There was no recurrence of TC after 1 year follow-up.

## 3. Discussion and conclusion

In 1969, Pinkus confirmed that TC originated from the outer hair root sheath of the isthmus of hair follicle, and the cells of the outer hair root sheath of hair follicle proliferated due to gene changes, but what kind of gene changes are still unclear. Seidenari et al^[3]^ think that PTCH1 gene may be related to the occurrence of this disease. TC, proliferative TC and malignant proliferative TC are different stages of the same disease. The World Health Organization’s Pathology and Genetics of Skin Tumours (WHO, 2006) listed them as lumps with hair follicle differentiation, and the pathological morphology was divided into a continuous morphological pedigree from benign to malignant. There are rare cases of malignant transformation in TC,^[4]^ and if malignant transformation occurs, it can lead to distant metastasis. TC lesions are often multiple, and there are a few single lesions.^[5–7]^ The lesions are located in the parts with dense hair follicles and vigorous hair growth. More than 90% of them are found in the scalp, and a few are located in vulva and bulbar conjunctiva. TC is more common in middle-aged people, especially in women.^[8]^ Clinically, the course of the disease develops slowly, and most of them are round, hard and protruding masses, with no lesions on the surface skin and hair. CT or ultrasound is feasible for auxiliary examination, and postoperative pathological examination is needed for diagnosis, and the prognosis is good.

TC has the following characteristics: The contents of the cyst tend to be eosinophilic hair keratin and its destruction products, and the form of keratosis is hairy keratosis and non-epidermal keratosis; That content of the cyst may bind to human hair-derived antibodies; 25% of the lesions showed calcification in the cyst. The lesions always have sheet or arenaceous calcification^[9]^; The epithelial cells of the cyst wall keratose rapidly and there is no granular layer^[10]^; The cyst has clear boundary with poor blood supply and solid nature without cystic degeneration under the naked eye; The patient has a family history of TC.^[11]^

This disease needs to be distinguished from other cysts: Epidermoid cyst: it occurs mainly in young people and children, but rarely in the scalp. The contents of the cyst can bind to antibodies derived from human corpus callosum.^[12]^ The cyst wall was composed of 2 layers of tissue, with the outer layer being fibrous connective tissue and the inner layer being stratified squamous epithelium with a granular layer. The basal lay is arranged on that out layer of the cyst wall, and the stratum corneum is arranged on the inner lay of the cyst wall; Dermoid cyst, which is often found in children, has cyst wall with skin adnexa such as sebaceous gland, hair follicle, hair and other germ layer structures in addition to squamous epithelium and fibrous tissue, belonging to the category of mature teratoma; The sebaceous cyst, commonly known as “pink tumor,” is caused by obstructed sebum excretion and is more common in young people.^[13,14]^ The cyst wall is composed of stratified squamous epithelium with focal sebaceous gland cells. TC progresses slowly, and the masses are small and small in number, which may justify conservative management, but further treatment is required in the event of ulceration and infection. In general, the treatment is based on complete surgical resection, and recurrence can be caused by residual cyst wall. In our patient, all the lumps were completely excised during the operation, and the wound healed well without recurrence after operation.

## Author contributions

**Conceptualization:** Zipeng Zhu.

**Investigation:** Zipeng Zhu, Xiaoqian Yang.

**Methodology:** Zipeng Zhu, Xiaoqian Yang.

**Project administration:** Zipeng Zhu.

**Resources:** Zipeng Zhu, Jinyue Fu.

**Supervision:** Zipeng Zhu, Shubao Zhang.

**Validation:** Zipeng Zhu, Shubao Zhang.

**Writing – original draft:** Zonglei Chong.

**Writing – review & editing:** Zonglei Chong.
